# Practical Aspects of Model-Based Collision Detection

**DOI:** 10.3389/frobt.2020.571574

**Published:** 2020-11-23

**Authors:** Shamil Mamedov, Stanislav Mikhel

**Affiliations:** ^1^Laboratory of Mechatronics, Control and Prototyping, Center for Technologies in Robotics & Mechatronics Components, Innopolis University, Innopolis, Russia; ^2^Industrial Robotics Lab, Center for Technologies in Robotics & Mechatronics Components, Innopolis University, Innopolis, Russia

**Keywords:** dynamic identification, collision detection, disturbance observer, human-robot interaction, observer design, physical human-robot interaction

## Abstract

Recently, with the increased number of robots entering numerous manufacturing fields, a considerable wealth of literature has appeared on the theme of physical human-robot interaction using data from proprioceptive sensors (motor or/and load side encoders). Most of the studies have then the accurate dynamic model of a robot for granted. In practice, however, model identification and observer design proceeds collision detection. To the best of our knowledge, no previous study has systematically investigated each aspect underlying physical human-robot interaction and the relationship between those aspects. In this paper, we bridge this gap by first reviewing the literature on model identification, disturbance estimation and collision detection, and discussing the relationship between the three, then by examining the practical sides of model-based collision detection on a case study conducted on UR10e. We show that the model identification step is critical for accurate collision detection, while the choice of the observer should be mostly based on computation time and the simplicity and flexibility of tuning. It is hoped that this study can serve as a roadmap to equip industrial robots with basic physical human-robot interaction capabilities.

## 1. Introduction

With the advancement and proliferation of robotics, particularly in the manufacturing industry, human-robot interaction (HRI) is becoming more complex. The safe collaboration of humans and robots is being studied within the physical human-robot interaction (pHRI) field, which considers such collaboration to be based upon the ability of robots to sense their environment. In its simplest form pHRI boils down to monitoring robots' collisions with the environment or humans and stopping it if a collision has been detected. A more sophisticated realization of pHRI in terms of software should include collision localization (Haddadin et al., 2017; Mikhel et al., 2019), collision reaction strategies (Haddadin et al., 2008), relevant control techniques such as force/impedance control, and real-time motion planning (De Santis et al., 2008). In this paper, we focus on a simple form of pHRI which can be used independently—in situations when a robot is performing a task and a human happens to be in its way, or in situations when a human, by deliberately coming into contact with a robot, can prevent it from hurting itself, others, or damage the environment—or as a part of more sophisticated pHRI used for supportive, collaborative, or cooperative interactions (Haddadin and Croft, 2016).

There are two main approaches to collision detection: model-free and model-based. Model-free methods usually compare torques/currents needed to execute the trajectory with real torques applied to the robot, and if a collision occurs, a certain threshold is exceeded (Takakura et al., 1989). These methods require access to the trajectory planner and controller of the robot; however, such access is not available to the users of the industrial manipulators. On the other hand, model-based methods use the dynamic model of a robot to estimate the disturbance torques using observers, and, based on the value of such torques, conclude the presence of a collision.

Most studies on model-based pHRI focus primarily on collision detection schemes or external torque estimation where they assume that the real model of the robot is known (Haddadin et al., 2017; Garofalo et al., 2019). However, systematic treatment of all three aspects of basic pHRI–model identification, observer design, and collision detection–and the relationship between the three has not been addressed yet. This study aims to bridge this gap and put forward a clear roadmap for enabling industrial robots with basic pHRI capabilities. The remaining part of the paper proceeds as follows: in the Model Identification subsection we outline the main steps needed for accurate dynamic parameters estimation; for each step we review relevant literature and provide practical advice. Compared to Wu et al. (2010) our literature review includes important recent contributions and is primarily intended for practitioners. In the Disturbance Estimation subsection, we review and compare several algorithms in terms of computation time as well as simplicity and flexibility of tuning. Our paper reviews alternative observers to the well-established pHRI community momentum observer and provides a new perspective of the momentum observer. In the Collision Detection subsection, we review both classical and recently proposed collision detection algorithms and discuss their advantages and limitations. Finally, we demonstrate the roadmap on a case study that involved manipulator UR10e.

## 2. Theoretical Preliminaries

Industrial manipulators might be subject to external disturbances due to collisions or contact with the environment. Usually, industrial manipulators are supplied with a controller and a trajectory planner. The majority of such manipulators allow users to input high-level tasks and measure torques/currents, positions, and velocities; however, such robots give users access to neither control nor the output of the trajectory planner. Given everything mentioned above, a question arises as to the most efficient way of detecting collisions of robot with their environment. In this section, we answer this question by consecutively discussing three main cornerstones that constitute model-based collision detection: model identification, observation, and detection.

### 2.1. Model Identification

An accurate dynamic model is the paramount element of any model-based collision detection algorithm. The manipulator dynamics equation generally used is

(1) $$\begin{array}{l} M\left(q\right)\ddot{q}+C\left(q,\dot{q}\right)\dot{q}+F\left(\dot{q}\right)+g\left(q\right)=\tau +\tau_{ext}, \end{array}$$

where $q,\;\dot{q},\;\ddot{q}\in {\mathbb{R}}^{n}$ are the vectors of generalized coordinates, velocities, and acceleration, respectively; **M**(***q***) ∈ ℝ^*n*×*n*^ is an inertia matrix; $C\left(q,\dot{q}\right)\in {\mathbb{R}}^{n\times n}$ is a matrix of Coriolis and centrifugal forces; $F\left(\dot{q}\right)\in {\mathbb{R}}^{n}$ and ***g***(***q***) ∈ ℝ^*n*^ are the vectors of friction and gravitational torques; ***τ*** is the vector of the actuation torques; ***τ***_*ext*_ is the vector of the torques induced at the joints by the contact forces, in the absence of contact/collision with the environment ***τ***_*ext*_ = **0** (Siciliano et al., 2010). The model requires the exact knowledge of the kinematic and dynamic parameters. In real life, kinematic parameters are known (because of manufacturing standards and calibration of robots before shipping) and provided by manufacturers. However, the latter usually do not provide dynamic parameters, with rare exceptions like, for example, Universal Robots that provide CAD models. Even with the CAD model the torque prediction lacks accuracy because it contains neither friction nor motor inertia parameters. A more accurate model can be obtained by performing identification. In this subsection, we provide an outline of the identification procedure.

The process of the identification of the dynamic parameters of mechanical systems can be divided into seven main steps (Swevers et al., 2007):

1. derivation of the dynamic model in regressor form
2. computation of base inertial parameters
3. trajectory planning
4. experiment conducting and collecting data
5. data processing
6. parameter estimation
7. validation

Identification starts by deriving the dynamic model of a manipulator moving in free space (***τ***_*ext*_ = **0**) in a linear regressor form

(2) $$\begin{array}{l} Y\left(q,\dot{q},\ddot{q}\right)\pi =\tau , \end{array}$$

where $Y\left(q,\dot{q},\ddot{q}\right)\in {\mathbb{R}}^{n\times n_{\pi }}$ is the regressor matrix, that can be obtained symbolically or numerically using recursive Lagrange-Euler formulation (Siciliano et al., 2010), modified recursive Newton-Euler formulation (Atkeson et al., 1986), or screw theory formulation (Garofalo et al., 2013), and $\pi \in {\mathbb{R}}^{n_{\pi }}$ is the vector of dynamic parameters (standard parameters). It consists of inertial parameters of each link modeled as a rigid body, an actuator, and friction parameters of each joint. The inertial parameters of a link include mass *m*_*i*_, the first moment of inertia ***h***_*i*_ = *m*_*i*_***r***_*i*_ (the product of a mass and the position of the center of mass), and inertia tensor with respect to the origin of the link ***I***_*i*_. Friction parameters are the coefficient of the Coloumb friction *f*_*c*_, the coefficient of the viscous friction *f*_*v*_, and an offset due to motor current offset *f*_*o*_. Actuator parameter is reflected rotor inertia *J*_*i*_ [If servomotor inertia *I*_*m, i*_ and the gear ratio of the transmission *k*_*ri*_ are known, then reflected rotor inertia can be computed as $J_{i}=I_{m,i}/k_{ri}^{2}$ and the components of the regressor corresponding to reflected inertia should be moved to the right-hand side of Equation (2)]. In total, the vector of dynamic parameters for each link is

(3) $$\begin{array}{l} \pi_{i}={\left[\begin{array}{cccccccccccc} I_{xx}^{i} & I_{xy}^{i} & I_{xz}^{i} & I_{yy}^{i} & I_{yz}^{i} & I_{zz}^{i} & h_{i}^{T} & m_{i} & J_{i} & f_{v}^{i} & f_{c}^{i} & f_{o}^{i} \end{array}\right]}^{T}\in {\mathbb{R}}^{14}. \end{array}$$

*Remark. More advanced friction models, for example, models with the Stribeck effect are not linear in parameters, yield a non-linear regressor matrix, that significantly complicates the identification process. However, if the linear friction model does not accurately describe friction torques, then it can be replaced by a non-linear model after identifying all other parameters. The non-linear model is usually identified for each link separately using non-linear least squares* (Gaz et al., 2018).

Not all the dynamic parameters enter the dynamic equation of the robot independently; some of them do not enter at all, some of them enter in linear combinations with other parameters. Due to that, the regressor matrix ***Y***(·) has zero or linearly dependent columns leading to singularity which causes problems during trajectory planning and parameter estimation. Several methods have been proposed to overcome these limitations by deriving a set of identifiable parameters called base parameters ***π***_*b*_ and corresponding base regressor matrix ***Y***_*b*_(·). Gautier and Khalil (1990) proposed an analytical method based on the recursive properties of robot energy. However, a year later Gautier (1991) proposed numerical methods based on QR- and SVD- decompositions. The analytical method was derived using Denavit–Hartenberg convention and it employs heuristics for determining linear dependence or independence of actuator parameters. Numerical methods (especially QR) are easy to implement and not limited to a specific convention, thus they should be the first choice when approaching parameter reduction.

The choice of trajectory significantly affects the accuracy of identification. For example, random point-to-point motion will most probably not lead to accurate parameter estimates because of the lack of property called persistence of excitation (Anderson, 1982). To find a persistently exciting trajectory it is necessary to carry out trajectory planning that is usually posed as an optimization problem (non-linear program) that aims to find $q_{*}\left(t\right),\;{\dot{q}}_{*}\left(t\right)$, and ${\ddot{q}}_{*}\left(t\right)$ such that chosen objective function associated with persistence of excitation is minimized. The most commonly used objective functions are condition number of the observation matrix [cond(***W***_*b*_), for the definition of ***W***_*b*_ see Equation (5)] or log-determinant of the moment matrix ($\text{log}\left(\text{det}\left(W_{b}^{T}W_{b}\right)\right)$) (Hollerbach et al., 2016). For industrial manipulators, several families of trajectories were proposed in the literature: fifth-order polynomials (Atkeson et al., 1986), truncated Fourier series (Swevers et al., 1997), and their combination (Wu et al., 2012). Fifth order polynomials have fewer parameters to optimize and can guarantee zero velocity and acceleration in the beginning and at the end of the trajectory. Periodic trajectories have an advantage in terms of data processing, as the same periodic trajectory can be executed several times and the collected data can be averaged. Moreover, they allow for exact frequency domain differentiation. However, there is a disadvantage in terms of abrupt change in initial velocities and accelerations (${\dot{q}}_{i}\left(0\right)\neq 0$, ${\dot{q}}_{i}\left(T\right)\neq 0$, ${\ddot{q}}_{i}\left(0\right)\neq 0$, ${\ddot{q}}_{i}\left(T\right)\neq 0$), which may cause robot vibration as well as hinder accurate trajectory tracking. Even in cases when zero initial and final velocities and accelerations are imposed as constraints of the optimization problem, the solver struggles to satisfy the constraints. The addition of a fifth-order polynomial solves this problem leading to the trajectory in the form

(4) $$\begin{array}{l} q_{i}\left(t\right)=\overset{N}{\underset{k=1}{\sum }}\left[\frac{a_{i,k}}{\omega_{f}k}\text{sin}\left(\omega_{f}kt\right)-\frac{b_{i,k}}{\omega_{f}k}\text{cos}\left(\omega_{f}kt\right)\right]\\ \begin{array}{l} \;+\overset{5}{\underset{i=0}{\sum }}c_{i,k}{\left(t-\left\lfloor \frac{t}{T}\right\rfloor T\right)}^{k}, \end{array} \end{array}$$

where *w*_*f*_ is the fundamental frequency, *N* is the number of harmonics, and ⌊ ⌋ is floor function.

Once trajectory planning has been performed, it is necessary to execute the trajectory on a real robot in closed-loop and record currents/torques, generalized positions ***q***(*t*), and, if available, generalized velocities $\dot{q}\left(t\right)$. After the trajectory execution, the recorded data should be processed to remove measurement noise and to reconstruct missing variables from the available ones. If $\dot{q}\left(t\right)$ are measurable, then only accelerations have to be estimated from velocities; otherwise, velocities have to be found first based on position measurements. As data processing is performed offline, the central difference scheme should be used for a more accurate estimation of derivatives (Hoffman and Frankel, 2018). However, derivative estimates are noisy even with a central difference scheme, so they should be filtered. To avoid introducing delays, non-casual filters (a zero-phase filter) should be used during filtering (Mitra and Kuo, 2006).

For parameter estimation, it is necessary to calculate observation matrix ***W***_*b*_(·) and observation vector ***T*** from the collected and processed data as

(5) $$\begin{array}{l} T=\left[\begin{array}{c} \tau \left(t_{1}\right)\\ ...\\ \tau \left(t_{k}\right)\\ ...\\ \tau \left(t_{n}\right) \end{array}\right],\;W_{b}=\left[\begin{array}{c} Y_{b}\left(q\left(t_{1}\right),\dot{q}\left(t_{1}\right),\ddot{q}\left(t_{1}\right)\right)\\ ...\\ Y_{b}\left(q\left(t_{k}\right),\dot{q}\left(t_{k}\right),\ddot{q}\left(t_{k}\right)\right)\\ ...\\ Y_{b}\left(q\left(t_{n}\right),\dot{q}\left(t_{n}\right),\ddot{q}\left(t_{n}\right)\right) \end{array}\right]; \end{array}$$

then, compute base parameters estimates employing ordinary least squares

(6) $$\begin{array}{l} {\hat{\pi }}_{b}={\left(W_{b}^{T}W_{b}\right)}^{-1}W_{b}^{T}T, \end{array}$$

which minimizes the squared torque prediction error, i.e., the cost function $J=\frac{1}{2}\|T-W_{b}\pi_{b}{\|}_{2}$. However, Equation (5) does not guarantee the physical consistency of parameters, thus the properties of the dynamic model (Siciliano et al., 2010). Since model-based techniques are extensively used in robotics, and the properties of dynamic equations are used to prove theorems and to guarantee convergence of controllers and observers, several important developments have been made in dynamic parameter identification in terms of the physical consistency of parameters. First, Sousa and Cortesão (2014) proposed to impose linear matrix inequality constraint on kinetic energy, more specifically—on the generalized inertia matrix of each link (semi-physical consistency)

(7) $$\begin{array}{l} \left[\begin{array}{cc} I_{i} & S\left(m_{i}r_{i}^{T}\right)\\ S\left(m_{i}r_{i}^{T}\right) & m_{i}I \end{array}\right]≻0, \end{array}$$

where ***S***(·) is the operator that maps a 3 × 1 vector into a 3 × 3 skew-symmetric matrix. Then, Wensing et al. (2017) and Sousa and Cortesao (2019) proposed to impose triangle inequality as the linear matrix inequality constraint (physical consistency)

(8) $$\begin{array}{l} \left[\begin{array}{cc} \frac{1}{2}tr\left(I_{i}\right)I-I_{i} & r_{i}\\ r_{i}^{T} & m_{i} \end{array}\right]≻0. \end{array}$$

Besides the constraints on inertial parameters of links, it is possible to impose constraints on other dynamic parameters: reflected inertia (*J*_*i*_ > 0) and friction ($f_{v}^{i}>0,\;f_{c}^{i}>0$). To impose physical or semi–physical consistency constraints while searching for base parameters, Sousa and Cortesão (2014) proposed a bijective mapping from the base and dependent parameters to standard parameters. It does not require additional computation, rather uses the result of base parameter calculation.

For some robots, instead of torque measurements, current measurements are available. In such cases, in addition to ***π*** (***π***_*b*_), it is necessary to estimate drive gains. For that, Gautier and Briot (2014) proposed to carry out an additional experiment with a load attached to the end-effector of the robot and use the extended observation vector ***T*** and matrix ***W***_*be*_

(9) $$\begin{array}{l} T=\left[\begin{array}{c} I_{u}\\ I_{l} \end{array}\right]K,\;W_{be}=\left[\begin{array}{ccc} W_{b} & 0 & 0\\ W_{b} & W_{lu} & W_{lk} \end{array}\right], \end{array}$$

where ***I***_*u*_ and ***I***_*l*_ are current measurements for unloaded and loaded cases respectively; ***K*** is a vector of drive gains; $W_{l}=\left[\begin{array}{cc} W_{lu} & W_{lk} \end{array}\right]\in {\mathbb{R}}^{10}$ is the observation matrix of the load, divided into parts corresponding to unknown and known inertial parameters. Using Equation (9), it is possible to derive the dynamics equation in a regression form linear with respect to unknown parameters

(10) $$\begin{array}{l} \left[\begin{array}{c} 0\\ W_{lk}\pi_{lk} \end{array}\right]=\left[\begin{array}{ccc} W_{b} & 0 & I_{u}\\ W_{b} & W_{lu} & I_{l} \end{array}\right]\left[\begin{array}{c} \pi_{b}\\ \pi_{lu}\\ K \end{array}\right], \end{array}$$

that allows estimating base dynamic parameters, unknown load inertial parameters, and drive gains, satisfying the (semi)-physical consistency constraints provided that some of the parameters of the load ***π***_*lk*_ are known (at least one), usually it is mass because it is easier to measure compared with other inertial parameters. Gautier and Briot (2014) proposed another method for computing drive gains using total least squares to avoid bias errors caused by the same $q\left(t\right),\;\ddot{q}\left(t\right)$, and $\ddot{q}\left(t\right)$ used in both parts of Equation (10). However, it is not clear how to impose constraints in that case; without constraints, such a computation method can result in negative drive gains.

*Remark. For drive gain identification, it is important to choose a load such that torques for loaded and unloaded trajectories differ. It can be achieved by choosing a heavy load and attaching it in a way that is not aligned with the axis of the end-effector*.

After the identification of parameters, model validation should be performed to assess the accuracy of torque prediction for any arbitrary trajectory executed by the robot. One example of a metric that can be used to measure accuracy is root mean square for the prediction error. If torque predictions are poor, then steps 3 to 6 should be repeated until the accurate model of the robot is obtained.

### 2.2. Disturbance Estimation

Ideally, to implement collision detection, it is necessary to estimate ***τ***_*ext*_, as it is the only indicator of the collision. However, even with the most recent model identification methods, it is impossible to reconstruct the exact model of a robot (Equation 1). The most accurate model available is

(11) $$\begin{array}{l} \hat{M}\left(q\right)\ddot{q}+\hat{C}\left(q,\dot{q}\right)\dot{q}+\hat{F}\left(\dot{q}\right)+\hat{g}\left(q\right)=\hat{\tau }+\tau_{ext}, \end{array}$$

with a mismatch between the real model and its estimate (unmodeled dynamics) being equal to

(12) $$\begin{array}{l} \tau_{um}=\Delta M\left(q\right)\ddot{q}+\Delta C\left(q,\dot{q}\right)\dot{q}+\Delta F\left(\dot{q}\right)+\Delta g\left(q\right). \end{array}$$

Using Equation (12) it is possible to rewrite Equation (11) as

(13) $$\begin{array}{l} \hat{M}\left(q\right)\ddot{q}+\hat{C}\left(q,\dot{q}\right)\dot{q}+\hat{F}\left(\dot{q}\right)+\hat{g}\left(q\right)=\tau +\tau_{d}. \end{array}$$

Thus, the parameter that can be estimated is lumped disturbance ***τ***_*d*_ = −***τ***_*um*_ + ***τ***_*ext*_. If variables ***q***, $\dot{q}$, $\ddot{q}$, ***τ*** can be measured, then the vector of disturbance torques can be found from Equation (13) through straightforward subtraction. In practice, this approach is hardly applicable, since acceleration measurements are not available, and estimating them from noisy velocity measurements through numerical differentiation further amplifies noise. Therefore, more sophisticated techniques should be considered that would allow for estimating ***τ***_*d*_ in absence of acceleration measurements. In the following two subsections we review several disturbance observers proposed in the context of pHRI.

#### 2.2.1. Non-linear Disturbance Observer (NDOB)

Inspired by the disturbance observer for linear systems, Chen et al. (2000)—first assuming that acceleration measurements are available—proposed an observer for lumped disturbance estimation

(14) $$\begin{array}{l} {\dot{\hat{\tau }}}_{d}=Le,\;e=\left(\tau_{d}-{\hat{\tau }}_{d}\right), \end{array}$$

where ***L*** ∈ ℝ^*n*×*n*^ is the observer gain matrix. In general, the error dynamics of the observer is

(15) $$\begin{array}{l} \dot{e}={\dot{\tau }}_{d}-Le, \end{array}$$

if there is no prior information on ***τ***_*d*_, then ${\dot{\tau }}_{d}$ is set to zero yielding

(16) $$\begin{array}{l} \dot{e}+Le=0. \end{array}$$

When ***L*** is a constant stable matrix, the estimation error tends to be zero. However, due to lack of acceleration measurements, the authors modified the original observer by introducing an auxiliary variable $z={\hat{\tau }}_{d}-\psi \left(q,\dot{q}\right)$ such that

(17) $$\begin{array}{l} \frac{d}{dt}\psi \left(q,\dot{q}\right)=L\hat{M}\left(q\right)\ddot{q}, \end{array}$$

while the dynamics of ***z*** is:

(18) $$\begin{array}{l} \dot{z}=-Lz+L\left[\hat{C}\left(q,\dot{q}\right)\dot{q}+\hat{F}\left(\dot{q}\right)+\hat{g}\left(q\right)-\tau -\psi \left(q,\dot{q}\right)\right]. \end{array}$$

(It is obtained by taking the time derivative of ***z*** followed by substituting Equation (14) for ${\dot{\hat{\tau }}}_{d}$ and Equation (13) for ***τ***_*d*_.) The special choice of $\psi \left(q,\dot{q}\right)$ used in modified observer results is the same error dynamics as the original observer (Equation 14).

The main difficulty in designing NDOB is choosing gain matrix ***L*** and vector $\psi \left(q,\dot{q}\right)$. Chen et al. (2000) proposed a design procedure for planar 2 degrees of freedom (DOF) manipulator, Nikoobin and Haghighi (2009) extended it to n-DOF planar manipulators. Later, Mohammadi et al. (2013) extended the observer to the spatial case, more specifically authors proposed to choose ***L*** and ***ψ*** as

(19) $$\begin{array}{l} \psi \left(\dot{q}\right)=X^{-1}\dot{q},\;L=X^{-1}\hat{M}{\left(q\right)}^{-1}. \end{array}$$

For ${\dot{\tau }}_{d}=0$ using candidate Lyapunov function $V=e^{T}X^{T}\hat{M}\left(q\right)Xe$ they proved that if ***X*** is invertible and there exists positive definite symmetric matrix **Γ** such that $X+X^{T}-X^{T}\dot{\hat{M}}\left(q\right)X\geq \Gamma$ then disturbance estimation error converges exponentially to zero (Mohammadi et al., 2013, Theorem 1). For ${\dot{\tau }}_{d}\neq 0$ if the rate of change of the lumped disturbance is bounded i.e., $\|{\dot{\tau }}_{d}\|\leq \kappa ,\;\kappa >0$, it was proven that the estimation error exponentially converges to a ball of a certain radius (Mohammadi et al., 2013, Theorem 2). In both cases, to design ***X*** the user can either solve linear matrix inequality

(20) $$\begin{array}{l} \left[\begin{array}{cc} Y+Y^{T}-\xi I & Y^{T}\\ Y & \Gamma^{-1} \end{array}\right]\geq 0, \end{array}$$

where ***Y*** = ***X***^−1^, ξ is upper bound of $\left\|\dot{M}\left(q\right)\right\|$ and ***I*** is identity matrix of proper dimension, or use analytical solution for the case when matrices ***Y*** and **Γ** are scaled identity matrices

(21) $$\begin{array}{l} Y=0.5\left(\xi +2\beta \sigma_{2}\right)I, \end{array}$$

where β is minimum convergence rate, σ is upper bound of inertia matrix (***M***(***q***) ≤ σ_2_***I***).

#### 2.2.2. Momentum Observer

Another widely used approach for disturbance estimation is based on generalized momentum and was proposed in the context of actuator fault detection and isolation (De Luca and Mattone, 2003). Below we show the derivation of the observer following the same steps as for NDOB. Given the definition of the momenta

(22) $$\begin{array}{l} p=M\left(q\right)\dot{q}, \end{array}$$

and its time derivative

(23) $$\begin{array}{l} \dot{p}=M\left(q\right)\ddot{q}+\dot{M}\left(q\right)\dot{q}\\ \begin{array}{l} \;=\tau +C^{T}\left(q,\dot{q}\right)\dot{q}-g\left(q\right)-F\left(\dot{q}\right)+\tau_{d}, \end{array} \end{array}$$

we can rewrite the disturbance dynamics defined in Equation (14) in terms of the rate of change of momentum

(24) $$\begin{array}{l} {\dot{\hat{\tau }}}_{d}=L\left(\dot{p}-\dot{\hat{p}}\right). \end{array}$$

If gain matrix ***L*** is constant, then by integrating both parts we can obtain a so-called momentum observer (Haddadin et al., 2017)

(25) $$\begin{array}{l} {\hat{\tau }}_{d}=L\left\{p-\int_{0}^{t}\left(\tau +{\hat{C}}^{T}\left(q,\dot{q}\right)\dot{q}-\hat{g}\left(q\right)-\hat{F}\left(\dot{q}\right)+{\hat{\tau }}_{d}\right)d\xi -p\left(0\right)\right\}, \end{array}$$

Compared with NDOB, momentum observer does not require inertia matrix inversion and each diagonal entry of the gain matrix ***L*** can be assigned independently.

Equation (23) can be interpreted as linear system

(26) $$\begin{array}{l} \dot{p}=0\;p+I\;u+\tau_{d}. \end{array}$$

where $u=\tau +C^{T}\left(q,\dot{q}\right)\dot{q}-g\left(q\right)-F\left(\dot{q}\right)$ and **0** is zero matrix of proper dimension. In Oh and Chung (1999), the authors used Equation (26) together with apriori information on disturbance–the disturbance is the output of some linear system (Johnson, 1971)

(27) $$\begin{array}{l} \dot{\omega }=G\omega ,\;\tau_{d}=F\omega , \end{array}$$

– to obtain extended linear system

(28) $$\begin{array}{l} \dot{\zeta }=\left[\begin{array}{cc} 0 & F\\ 0 & G \end{array}\right]\zeta +\left[\begin{array}{c} I\\ 0 \end{array}\right]u\\ \begin{array}{l} y=\left[\begin{array}{cc} I & 0 \end{array}\right]\zeta , \end{array} \end{array}$$

where ***ζ*** = ${\left[\begin{array}{cc} p^{T} & \omega^{T} \end{array}\right]}^{T}$ is the extended state vector. Then Oh and Chung (1999) used a reduced state observer in order to estimate ***τ***_*d*_. Hu and Xiong (2017) assumed that both states and output of Equation (28) are affected by white Gaussian noise and used the Kalman filter to estimate ***τ***_*d*_.

#### 2.2.3. Sliding Mode Momentum Observer

Garofalo et al. (2019) proposed a second-order sliding mode (Super Twisting Algorithm, Fridman and Levant, 2002) extension of the classical momentum observer:

(29) $$\begin{array}{l} \dot{\hat{p}}=u-T|\hat{p}-p{|}^{\frac{1}{2}}sgn\left(\hat{p}-p\right)+\sigma \\ \begin{array}{l} \dot{\sigma }=-S\;sgn\left(\hat{p}-p\right), \end{array} \end{array}$$

where ***S***, ***T*** ∈ ℝ^*n*×*n*^ are positive definite diagonal matrices with diagonal elements satisfying conditions given in Moreno and Osorio (2008) that guarantees global finite-time stability of the equilibrium point ${\left[\begin{array}{cc} \hat{p}-p & \sigma -\tau_{d} \end{array}\right]}^{T}=0$. Convergence property of the second order sliding mode observer can be improved—from finite time to exponential—by introducing linear correction terms (Moreno and Osorio, 2008; Garofalo et al., 2019):

(30) $$\begin{array}{l} \dot{\hat{p}}=u-T_{1}|\hat{p}-p{|}^{\frac{1}{2}}sgn\left(\hat{p}-p\right)-T_{2}\left(\hat{p}-p\right)+\sigma \\ \begin{array}{l} \dot{\sigma }=-S_{1}\;sgn\left(\hat{p}-p\right)-S_{2}\left(\hat{p}-p\right), \end{array} \end{array}$$

A sliding mode momentum observer is superior to a classical momentum observer in terms of noise attenuation and convergence time; however, it requires tuning many more parameters.

#### 2.2.4. Filtered Dynamics Observer

A different approach to disturbance estimation was put forward in Van Damme et al. (2011) and Ho and Song (2013) where they used the idea from online dynamic parameter estimation: if both parts of Equation (13) are filtered with a strictly stable filter then there is no need for acceleration measurements. To outline the derivation, assume we choose a filter with a transfer function

(31) $$\begin{array}{l} F\left(s\right)=\frac{1}{s/\omega +1}, \end{array}$$

and impulse response $f\left(t\right)=L^{-1}\left(F\left(s\right)\right)=\omega \text{ exp }\left(-\omega t\right)$. Multiplying both parts of Equation (13) by Equation (31) is equivalent to

(32) $$\begin{array}{l} f\left(t\right)*\left\{\hat{M}\left(q\right)\ddot{q}+\hat{C}\left(q,\dot{q}\right)\dot{q}+\hat{F}\left(\dot{q}\right)+\hat{g}\left(q\right)\right\}=f\left(t\right)*\left\{\tau +\tau_{d}\right\}, \end{array}$$

where * is used to denote convolution operation. Using properties of the convolution operation, we can rewrite Equation (32) as

(33) $$\begin{array}{l} f\left(t\right)*\left\{\tau +\tau_{d}\right\}=\dot{f}\left(t\right)*\left\{\hat{M}\left(q\right)\dot{q}\right\}+f\left(0\right)\hat{M}\left(q\right)\dot{q}-f\left(t\right)\hat{M}\left(q\left(0\right)\right)\dot{q}\left(0\right)\\ \begin{array}{l} \;+f\left(t\right)*\left\{\hat{C}\left(q,\dot{q}\right)\dot{q}+\hat{F}\left(\dot{q}\right)+\hat{g}\left(q\right)-\dot{\hat{M}}\left(q\right)\dot{q}\right\}, \end{array} \end{array}$$

where $\dot{f}$(*t*) is the impulse response of a stable filter

(34) $$\begin{array}{l} F_{2}\left(s\right)=-\frac{\omega^{2}}{s+\omega }, \end{array}$$

Rearranging Equation (33) and using properties of the rigid body dynamics, it is possible to obtain an expression for filtered disturbance

(35) $$\begin{array}{l} f\left(t\right)*\tau_{d}=\dot{f}\left(t\right)*\left\{p\right\}+\omega p+f\left(t\right)*\left\{\hat{F}\left(\dot{q}\right)+\hat{g}\left(q\right)-{\hat{C}}^{T}\dot{q}-\tau \right\}, \end{array}$$

(For detailed derivation see Lewis et al., 2003 Chapter 6.6 or Van Damme et al., 2011). Van Damme et al. (2011) showed that momentum observer and filtered dynamics observer are equivalent for special choice of ***L*** in Equation (25): all diagonal entries are the same.

### 2.3. Collision Detection

From Equation (13) we know that disturbance observers estimate the sum of torques due to unmodeled dynamics and interaction. An easy and effective way to detect collisions in presence of unmodeled dynamics is to set a static threshold (upper and lower bounds on unmodeled dynamics). The value of the threshold can be chosen as a percentage from maximum torque, for example, ±0.1τ_*max*_ (Haddadin et al., 2008) or determined from data used for validation of dynamic parameters. However, inaccurate parameter identification or use of dynamic parameters from CAD may result in a high threshold yielding long collision detection time. To deal with it several solutions were proposed in the literature.

Hu and Xiong (2017) proposed to improve the dynamic model by training a neural network (multi-layer perceptron) to predict the residual between measured torques and torques found from a rigid body model. As the input of the network, they used generalized positions and velocities that, according to authors, provide the same degree of accuracy as the network with generalized positions, velocities, and accelerations. Rigid body model enhanced with neural network allowed authors to choose tight thresholds thus decrease collision detection time.

Ho and Song (2013) proposed using a 2nd order band-pass filter in Equation (33) instead of a low-pass filter (Equation 31). By carefully choosing cut-off frequencies they were able to filter out low-frequency torques due to the motion of the robot and high-frequency torques due to noise, leaving torques caused by the collision. Band-pass filtering allowed authors to decrease threshold 5-fold compared to classical momentum observer yielding to the detection of collision in 5–6 ms. However, their approach is limited to cases when the dynamics of collision is significantly faster than the dynamics of the task, moreover, band-pass filtering modifies estimated disturbance torques making them impossible to use, for example, in collision localization. Haddadin et al. (2008) and Li et al. (2019) proposed to use two observers, one to detect slow or soft collisions using low-pass filtered collision torques and one to detect fast collisions using band-pass filtered collision torques.

Sotoudehnejad et al. (2012) proposed using time-variant thresholds that take into account uncertainties in inertial parameters of the robot as well as friction parameters. To estimate uncertainties authors proposed to conduct time-consuming experiments with a robot applying various collision torque at different states. Although it sounds promising, the difference in the detection time between the time-variant threshold and constant threshold is of the order of 0.1–0.01 s. Briquet-Kerestedjian et al. (2019) proposed to also consider uncertainties due to numerical differentiation used to find generalized velocities and acceleration from noisy encoder measurements. Their approach is different from Sotoudehnejad et al. (2012) in a way they treat the model of the manipulator. Briquet-Kerestedjian et al. (2019) consider a decentralized linear model and treats non-linear coupling between joints as a disturbance. It allows authors to use linear estimation techniques such as Kalman filter. Sotoudehnejad et al. (2012) on the other hand consider a centralized non-linear model of the manipulator and use the momentum observer. In general, it is not clear if time-consuming experiments worth the amount of time gained in collision detection time and if the time-variant threshold is robust and provide consistent result in the whole workspace of the robot.

## 3. Case Study: UR10e

In this section, we demonstrate all the steps required to implement model-based collision detection on real robot UR10e which is a collaborative industrial robot arm from the Universal Robots company. It weighs 33.5 kg, has a 10 kg payload, and the radius of workspace up to 1,300 mm. The robot consists of six rotating joints which allow having the full degree of freedom in Cartesian space.

### 3.1. Software Implementation

Within the scope of this work, we developed a C++ library for processing the measurements and estimating disturbance torques (https://github.com/mikhel1984/ext_observer). The library consists of two basic abstract classes: robot and observer. The former provides an interface for defining the dynamic model of a robot. The latter allows working with an observer regardless of its internal implementation: it defines an obligatory method that takes current joint angles, velocities, torques, and time step, and returns the disturbance torque estimate. The library can be included in the source code of a robot control program, used with MATLAB (loaded as a C shared library) or robot operating system (ROS) as a standalone component.

To start working with the library, a user has to create a new robot instance inheriting the basic abstract class and define its dynamic model. The dynamic model can be supplied in two forms: functions for $\hat{M}\left(q\right)$, $\hat{C}(q,\dot{q})$, $\hat{F}\left(\dot{q}\right)$, and $\hat{g}\left(q\right)$ generated based on symbolically derived model; numerical procedure such as recursive Newton-Euler algorithm (RNEA) implemented as ${rnea}_{g}\left(q,\dot{q},\ddot{q}\right)$ where subscript *g* indicates the value of the gravity constant. If the dynamics of the robot is computed numerically, the problem arises related to the computation of $\hat{C}{\left(q,\dot{q}\right)}^{T}$ used in the majority of the observers. One way to overcome this problem is to use the modified Newton-Euler algorithm (De Luca and Ferrajoli, 2009), another is to use the numerical approximation of the time derivative of the inertia matrix ($\dot{\hat{M}}\approx \Delta \hat{M}/\Delta t$). Even though the second method is easier to implement, its accuracy depends on sampling time Δ*t*. We propose a simple way to improve the accuracy, by using the definition of the rate of change of the inertia matrix

(36) $$\begin{array}{l} \dot{\hat{M}}=\left[\frac{\partial \hat{M}}{\partial q_{1}}\ldots \frac{\partial \hat{M}}{\partial q_{n}}\right]\dot{q}, \end{array}$$

where $\partial {\hat{M}}_{i}/\partial q_{i}\approx \Delta \hat{M}/\Delta q_{i}$ has to be calculated numerically, for example, with finite differences. Further, noting that all the observers we have discussed utilize the product ${\hat{C}}^{T}$ and $\dot{q}$ rather than ${\hat{C}}^{T}$, it is possible to reduce computational costs by evaluating the product directly as $\dot{\hat{M}}\dot{q}-\hat{C}\dot{q}$ or

(37) $$\begin{array}{l} {\hat{C}}^{T}\dot{q}\approx \overset{n}{\underset{i=1}{\sum }}\frac{1}{\Delta q_{i}}\left({rnea}_{0}\left(q+\Delta q_{i},0,\dot{q}\right)-p_{0}\right)-{rnea}_{0}\left(q,\dot{q},0\right), \end{array}$$

where $p_{0}={rnea}_{0}\left(q,0,\dot{q}\right)$ and *n* is the number of joints. From a computational point of view Equation (37) is cheaper than computing $\dot{\hat{M}}$ and $\hat{C}$ separately because it allows avoiding multiple calls of ***rnea***_*g*_(·) for column-wise evaluation of the matrix $\dot{\hat{M}}$.

Each observer has to be initialized when called for the first time; we suggest initializing based on the assumption that there is no collision, and disturbance torques are equal to zero. Moreover, each observer depends on its previous states that can be implicit as in the case of filters, or explicit and implemented via the integration of some parameters. Therefore, observer does not work as a “pure function” and cannot be used several times during a time instant.

#### 3.1.1. NDOB

NDOB is among the easiest in terms of implementation, partially because it does not require $C{\left(q,\dot{q}\right)}^{T}\dot{q}$. The dynamics of the auxiliary variable (Equation 18) can be stiff for high convergence rates, therefore, to integrate it, we used the implicit Euler method. As for initialization, since the output is ${\hat{\tau }}_{d}=z+\psi \left(q,\dot{q}\right)$, the initial value can be set to $z\left(0\right)=-\psi \left(q,\dot{q}\right)$. In terms of performance, NDOB was among the slowest (**Table 3**) because of the matrix inversion, to decrease computation time associated with it the properties of the inertia matrix have to be exploited.

#### 3.1.2. Momentum Observer

The observer retains states of the integrator $\int_{0}^{t}\left(\tau +{\hat{C}}^{T}\left(q,\dot{q}\right)\dot{q}-\hat{g}\left(q\right)+{\hat{\tau }}_{d}\right)d\xi$, that can be initialized with zeros, but it is better to use −*p*(0) to avoid subtraction during the next calls. The efficiency of the observer depends on the accuracy of integration; a comparison of different integration schemes showed that a simple trapezoidal rule is sufficient for high sampling rates.

#### 3.1.3. Sliding Mode Momentum Observer

Both observers Equation (29) and Equation (30) can be implemented in single program code, as they only differ in coefficients ***T***_2_, ***S***_2_ that can be set to zero for second-order sliding mode observer. In our implementation, the dynamics of the observers is integrated using the explicit Euler method, and *sgn*(·) is replaced with the hyperbolic tangent to remove chattering. To initialize, $\hat{p}\left(0\right)$ can be set to ***p***(0).

#### 3.1.4. Kalman Filter Observer

The method evaluates the variable ***u*** and calls the implementation of the discrete-time Kalman filter. The filter requires the user to define the discrete-time model of the system (Equation 28). If the sampling rate is constant, then the discrete-time model can be calculated from the original continuous-time model offline. However, if sampling time varies then online matrix exponent computation has to be used. For a high sampling rate, we approximated matrix exponential by the first terms of its Taylor series expansion.

#### 3.1.5. Filtered Dynamics Observer

The implementation of the observer is based on infinite impulse response filters. In particular, *F*(*s*) and *F*_2_(*s*) are implemented as *y*_*i*_ = *k*_1_*y*_*i*−1_ + *k*_2_(*x*_*i*_ + *x*_*i*−1_) where *x* and *y* are the input and output respectively, *k*_1_ and *k*_2_ are coefficients of the filters. Both coefficients depend on the cut-off frequency of the filter and can be found, for example, via bilinear transforms. For disturbance estimation, *F*(*s*) can be initialized as $\hat{g}\left(q\right)-{\hat{C}}^{T}\dot{q}-\tau$, while *F*_2_(*s*) as ***p***.

### 3.2. Identifying Dynamic Model

Universal Robots provides a description of the UR10e in the universal robot description file (URDF). The file contains kinematic parameters of the robot as well as inertial parameters of links but does not contain inertial parameters of the motors, drive gains, and friction parameters needed for accurate modeling of dynamics. Thus, we performed model identification, following the main steps from section 2.1. First, we symbolically derived regressor matrix ***Y***(·) ∈ ℝ^6 ×84^ using kinematic parameters from URDF and Euler-Lagrange formulation of dynamics. Then reduced parameter space using QR—decomposition obtaining $\pi_{b}\in {\mathbb{R}}^{40}$ and $Y_{b}\left(\cdot \right)\in {\mathbb{R}}^{6\times 40}$.

**Figure 1:**
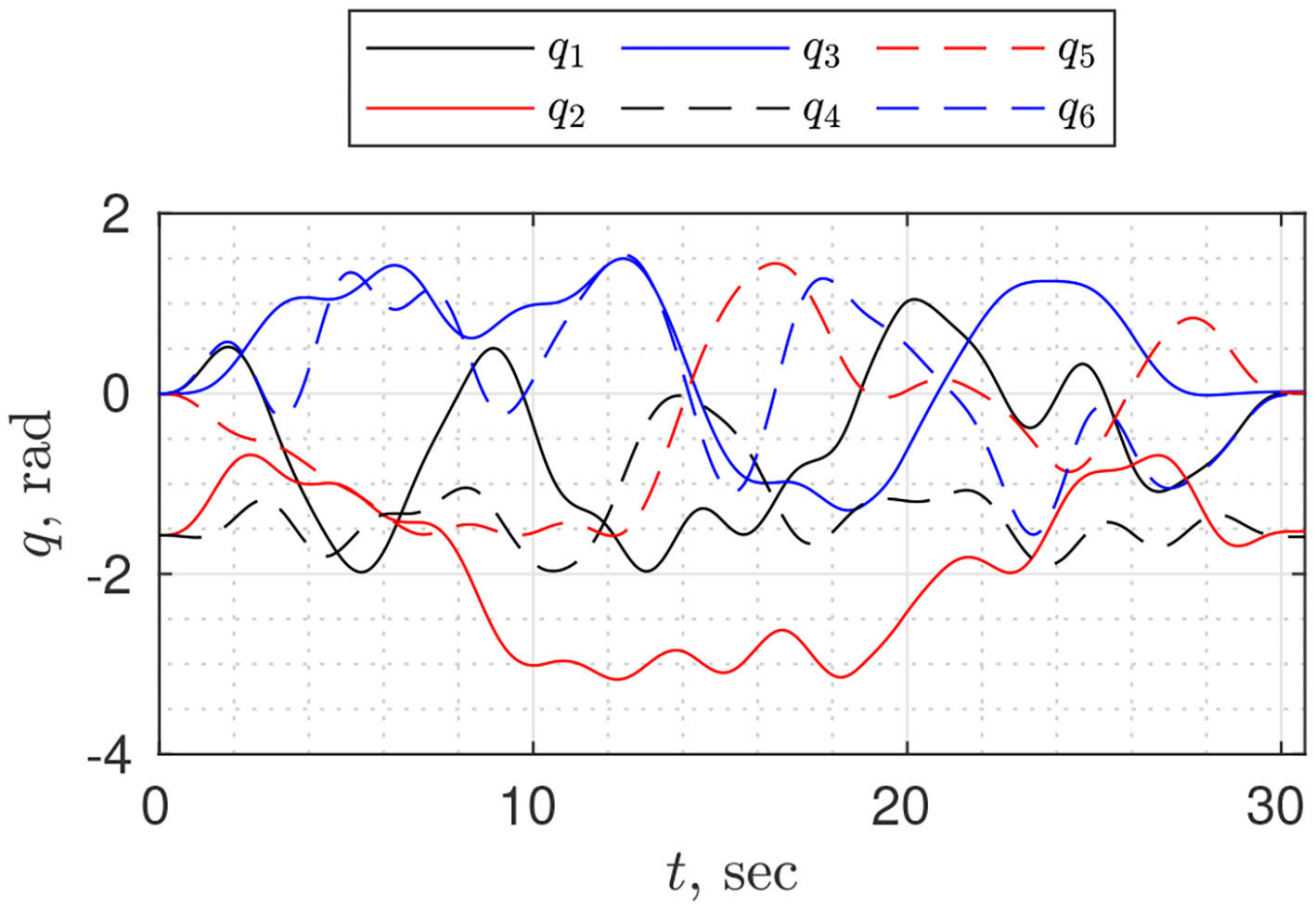
Optimized trajectory for dynamic parameter identification, executed on the robot UR10e. The condition number of the base observation matrix is *cond*(***W***_*b*_(·)) ≈ 93.27.

In experiment design, as a trajectory, we chose the combination of truncated Fourier series and fifth-order polynomials (Equation 4); as an objective function—condition number of the observation matrix. The optimization problem was posed in MATLAB and solved using the *patternsearch* algorithm. We performed trajectory optimization for different periods *T* (20, 30, 40, 50 s) and number of harmonics *N* (5, 8, 10, 12, 15); the best estimation in terms of root mean square error of torque prediction was obtained for *N* = 12 and *T* = 30 (Figure 1).

The optimized trajectory was executed on UR10e in the velocity control mode. There are several methods to program the robot to execute the desired trajectory: MATLAB, ROS, UR Script. We used UR Script because it allows sending commands to the robot with a higher sampling rate than other methods. Despite the trajectory tracking capabilities of the UR10e, the nominal and real trajectories do not always coincide because of safety limitations on velocities and accelerations. To overcome that either safety constraints can be removed, or maximum velocity and acceleration constraints can be decreased during the trajectory planning phase.

In the data processing stage currents, positions, and velocities were filtered with zero-phase fifth-order Butterworth filter (the *filtfilt* function in MATLAB). For acceleration estimation, we used numerical differentiation—central difference scheme—followed by zero-phase filtering to remove the noise.

**Figure 2:**
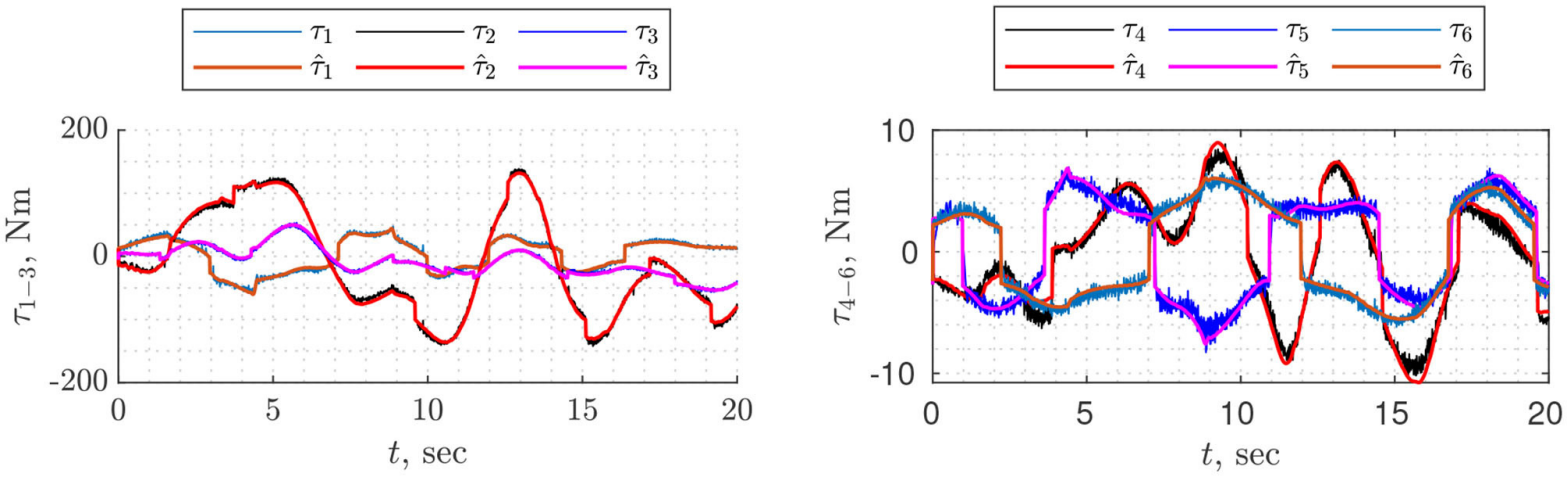
Validation of identified dynamic parameters. τ_*i*_ is measured current multiplied by identified drive gains *k*_*i*_, ${\hat{\tau }}_{i}$ estimated torque based on identified dynamic paramters.

Parameter estimation was divided into two parts: first, we identified drive gains on one trajectory (*T* = 50, *N* = 14), then on a different trajectory (*T* = 30, *N* = 12) the vector of base and standard parameters. In both cases, physical consistency was imposed by linear matrix inequality constraints using the YALMIP toolbox (Lofberg, 2004). Finally, the parameters were validated on a different harmonic trajectory (Figure 2) for which the root mean square error of torque prediction is *rms* = $\left[\begin{array}{cccccc} 2.81 & 4.16 & 1.90 & 0.70 & 0.65 & 0.46 \end{array}\right]\;Nm$. The estimated parameters are shown in Tables 1, 2.

**Table 1:** Identified link parameters.

	***I*_*xx*_**	***I*_*xy*_**	***I*_*xz*_**	***I*_*yy*_**	***I*_*yz*_**	***I*_*zz*_**	***h*_*x*_**	***h*_*y*_**	***h*_*z*_**	***m***
***No***	***kg* · *m*^2^**	***kg* · *m*^2^**	***kg* · *m*^2^**	***kg* · *m*^2^**	***kg* · *m*^2^**	***kg* · *m*^2^**	***kg* · *m***	***kg* · *m***	***kg* · *m***	***m***
1	16.02	0	0	16.02	0	10^−6^	0	0	0	4.83
2	3.93	0.85	0.15	3.72	−0.59	1.90	0.05	−0.28	3.26	7.93
3	2.22	−0.53	−0.18	1.39	−0.22	1.39	0.02	0.24	0.89	2.48
4	1.03	0.23	−0.01	0.08	0.07	1.07	−10^−3^	−0.25	0.19	2.16
5	0.03	−0.03	−0.03	0.11	−0.01	0.11	0.01	0.09	0.11	2.16
6	0.03	−10^−3^	−0.01	0.04	−10^−3^	0.01	10^−3^	−10^−3^	−0.02	0.22

**Table 2:** Identified motor and friction parameters.

	***K***	***J***	***f*_*v*_**	***f*_*c*_**	***f*_0_**
***No***	***N* · *m* · *A*^−1^**	***kg* · *m***	***N* · *s***	***N* · *m***	***N* · *m***
1	10.0	0	21.25	12.54	0.20
2	10.70	3.74	20.22	13.27	−0.75
3	8.46	0	10.38	4.99	0.20
4	9.0	0.07	3.58	2.0	0.05
5	9.48	0.23	2.49	2.69	−0.01
6	10.12	0.44	3.03	2.30	0.04

### 3.3. Choosing and Tuning Observers

In this subsection we provide the general guideline for selecting and tuning the disturbances observer discussed in the section 2.2. All the observers in absence of collisions show non-zero disturbance torques because of the inaccurate estimates of some dynamic parameters or unmodeled dynamics. The closer identified parameters are to real ones, the smaller are going to be estimated disturbance torques and vice versa. It emphasizes the significance of the accurate dynamic model in estimating torques associated with collisions. For high level comparison of the observers see Table 3.

**Table 3:** Comparison of observers in terms of computation time (average time needed to predict disturbance torques at a given time instant, laptop parameters: CPU Core i3 2.30GHz, RAM 4 GB) and tuning.

	**Average time**	***Tuning***	
	***ms***	***Simplicity***	***Flexibility***
Momentum observer	0.028	✓	✓
Nonlinear disturbance observer	0.146	✓	✓
Sliding mode observer	0.032	✗	✓
Kalman disturbance observer	0.294	✗	✓
Filtered dynamics	0.028	✓	✗

#### 3.3.1. NDOB

**Figure 3:**
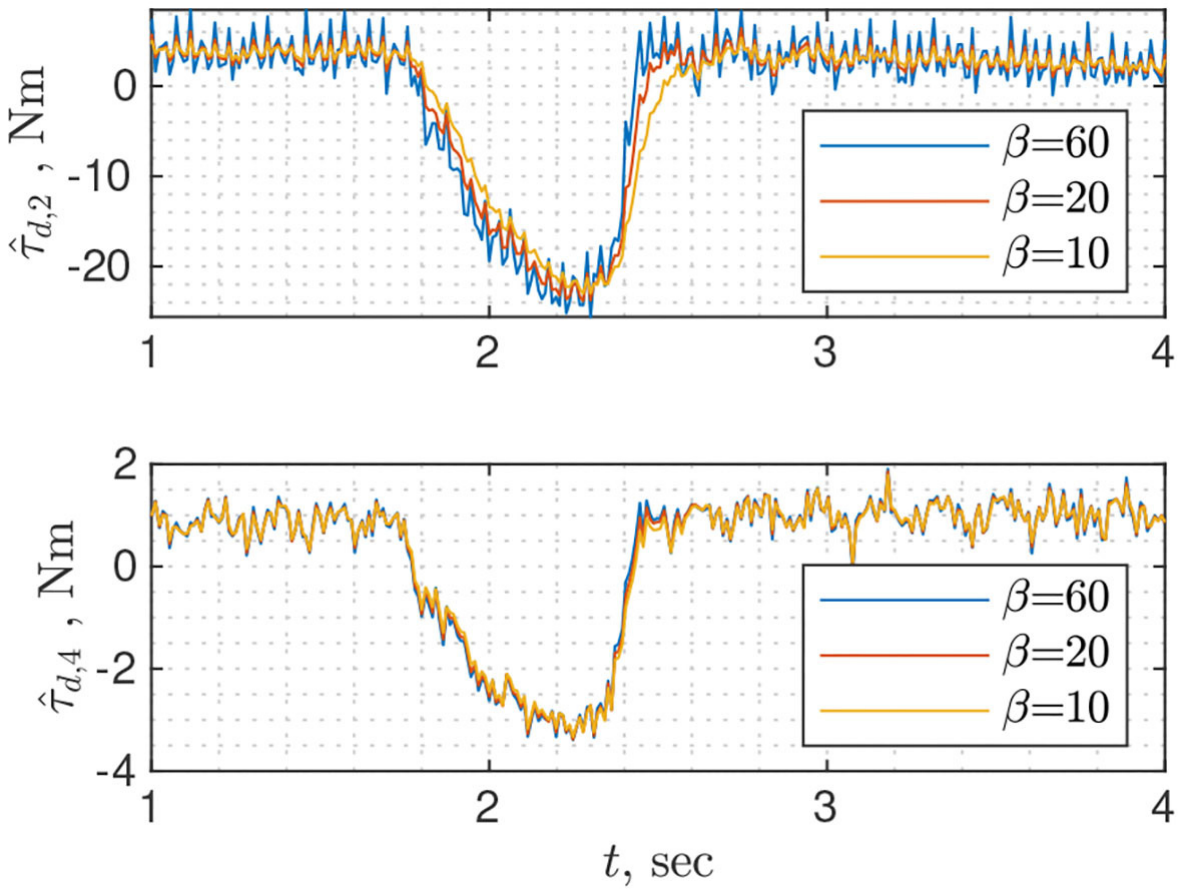
Disturbance torque estimation for joints 2 and 4 using nonlinear disturbance observer with different rates of convergence β. The change in τ_*d*_ is due to collision with the wrist.

To use NDOB, a user needs to specify gain matrix ***L*** and vector ***ψ***, both of which can be obtained from ***Y*** = ***X***^−1^ (Equation 19). There are two approaches to finding ***Y***: solving linear matrix inequality for a given positive definite symmetric matrix **Γ** (Equation 20); using analytical solution found for the case when **Γ** and ***Y*** are constrained to be scaled identity matrices (Equation 21). The latter is easier from a user perspective because it requires tuning single parameter β that defines minimum convergence rate whereas ξ and σ_2_ are constants that depend on the dynamics of the robot. While tuning the NDOB, whether matrix **Γ** or scalar β, the user should seek a compromise between convergence rate and measurement noise amplification. Figure 3 shows disturbance torque estimation for different values of β. For the second joint the lower is β the less noisy is ${\hat{\tau }}_{d,2}$, but for the fourth joint the value of β does not affect the amount of noise. It is the drawback of the analytical solution that all diagonal elements of ***Y*** are identical, thus the convergence rate of each joint cannot be tuned separately. Solving linear matrix inequality instead of using an analytical solution can serve as a remedy to this problem.

#### 3.3.2. Momentum Observer

**Figure 4:**
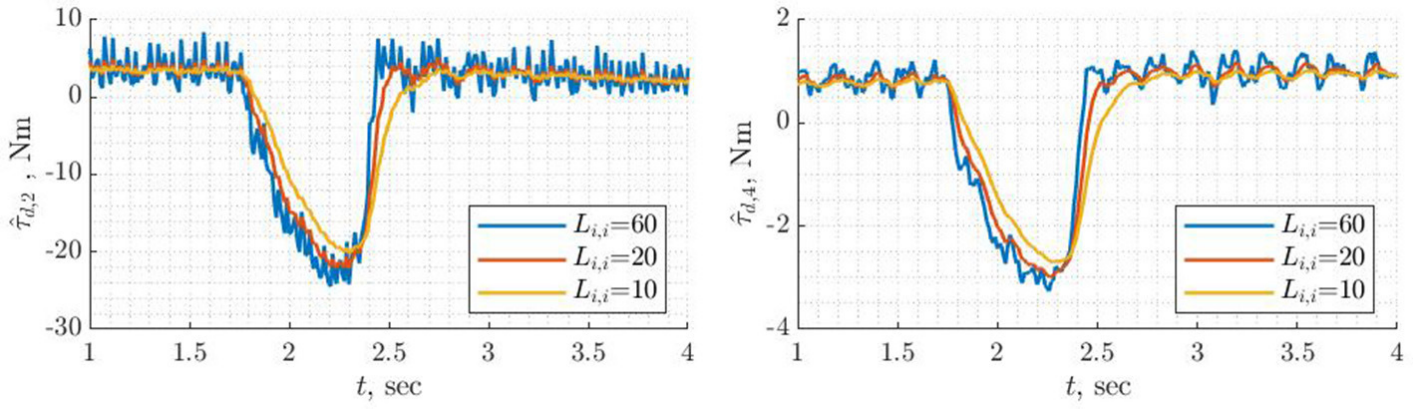
Disturbance torque estimation for joints 2 and 4 using classical momentum observer with different *L*(4, 4). The change in τd is due to collision with the wrist.

If ***L*** in Equation (25) is chosen as diagonal, then the classical momentum observer can be interpreted as *n* low-pass filters driven by disturbance torque of each joint (Figure 4). Therefore, the user can conveniently design each filter independently. On the other hand, the momentum observer can be treated as a linear time-invariant system (Equation 28) where disturbance torques are non-measurable states. In that case, the classical state observer can be designed using pole placement or linear quadratic techniques (Åström and Murray, 2010). The former is easier because the user needs to specify poles i.e., 12 parameters but it is not optimal, while the latter is more difficult to design as the user needs to tune matrices ***Q*** ∈ ℝ^12×12^ and ***R*** ∈ ℝ^6×6^ but it is optimal.

**Figure 5:**
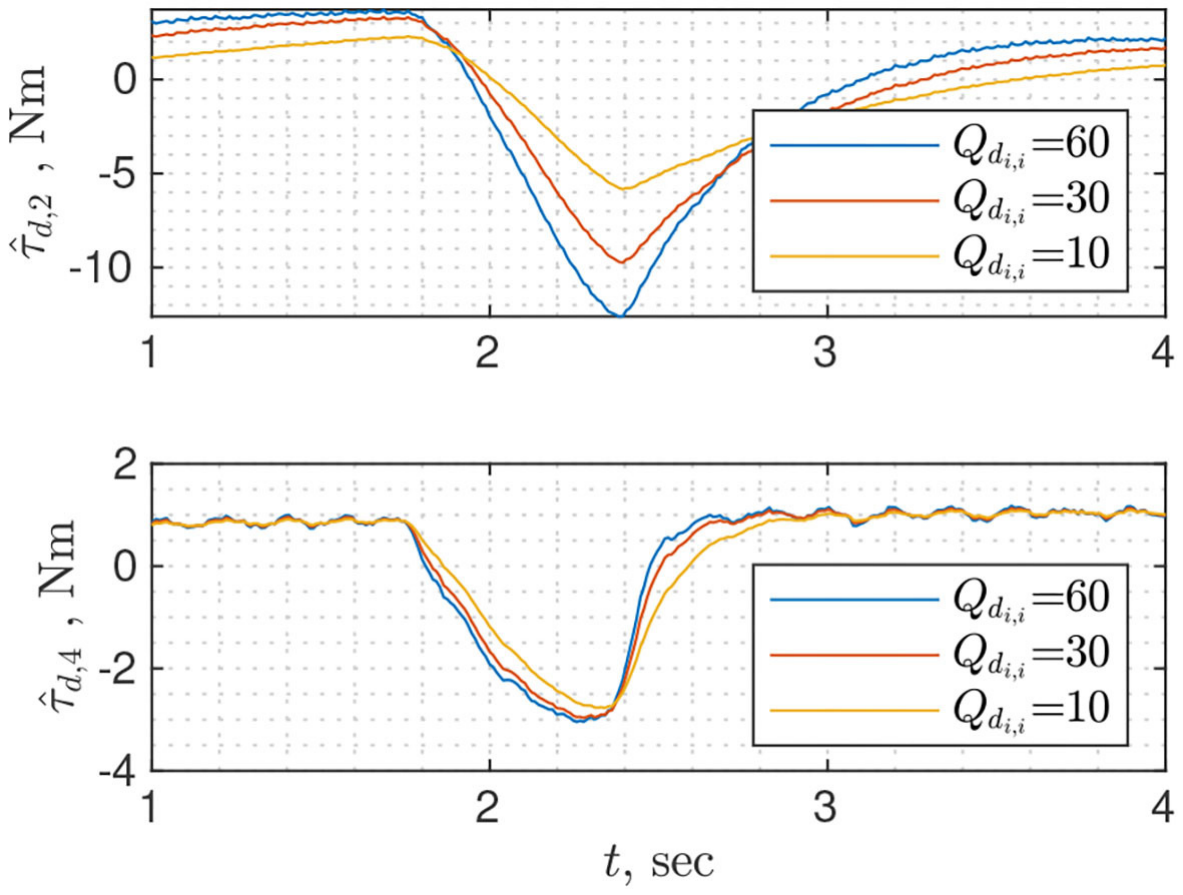
Disturbance torque estimation for joints 2 and 4 using Kalman disturbance observer with different disturbance covariance matrices.

If unmodeled dynamics is mainly due to friction modeling and can be described as zero-mean Gaussian white noise, then theoretically we can estimate ***τ***_*ext*_ by filtering out unmodeled dynamics using the Kalman filter. The price for accuracy is a time-consuming tuning procedure. To design the Kalman filter user needs to identify the diagonal covariance matrix of the unmodeled dynamics ***Q***_τ_*um*__ by individually moving each joint and calculating the variance of the $\tau_{um}^{i}$, then the diagonal covariance matrix of output measurement ***R***_*p*_ which is assumed to be mainly due to noise in velocity measurement. In turn, the covariance of the velocity measurement $R_{\dot{q}}$ can be found by moving each joint at a constant velocity and finding the variance of the residual $\Delta {\dot{q}}_{i}={\dot{q}}_{i}-{\bar{\dot{q}}}_{i}$. Then time-variant covariance matrix of the momentum can be computed by applying linear transformation properties of the normal $R_{p}=\hat{M}R_{\dot{q}}{\hat{M}}^{T}$. The only parameter that has to be tuned is the covariance matrix of the external torque dynamics. In the absence of any apriori information, it can be chosen the diagonal. The general guideline is that the larger diagonal elements are the less filter trusts the dynamics and the more amplifies noise. The interested reader is referred to Wahrburg et al. (2017) for more details. Figure 5 shows ${\hat{\tau }}_{d}$ for three different values of tuning parameter, the estimates are much less noisy and slower even for high values of the tuning parameter compared with other observers. Moreover, it does not filter model noise, probably because the assumptions on the diagonal structure of the covariance matrix as well as the source of unmodeled dynamics are too strong.

#### 3.3.3. Sliding Momentum Observer

The sliding mode observers are the most difficult to tune as they require tuning two matrices for second-order sliding mode observer and four matrices for second-order slide mode observer with linear terms matrices. Even if the matrices are restricted to be diagonal, the user needs to tune 12 and 24, parameters respectively for 6-DOF industrial manipulator. In order to guarantee the stability of the observers, the parameters should satisfy certain constraints (Moreno and Osorio, 2008). To obtain the desired behavior of the observer Garofalo et al. (2019) suggested considering the observer as a non-linear proportional-integral controller with all underlying tuning procedure. More specifically, they suggest tuning ***S***, then starting from $T=1.5\sqrt{S}$ tune ***T***. Linear terms of the second orders sliding mode observer with linear terms can be interpreted as second-order linear filter that allows obtaining the desired damping. For a more detailed discussion of tuning sliding mode momentum observers, readers are referred to Garofalo et al. (2019).

#### 3.3.4. Filtered Dynamics Observer

**Figure 6:**
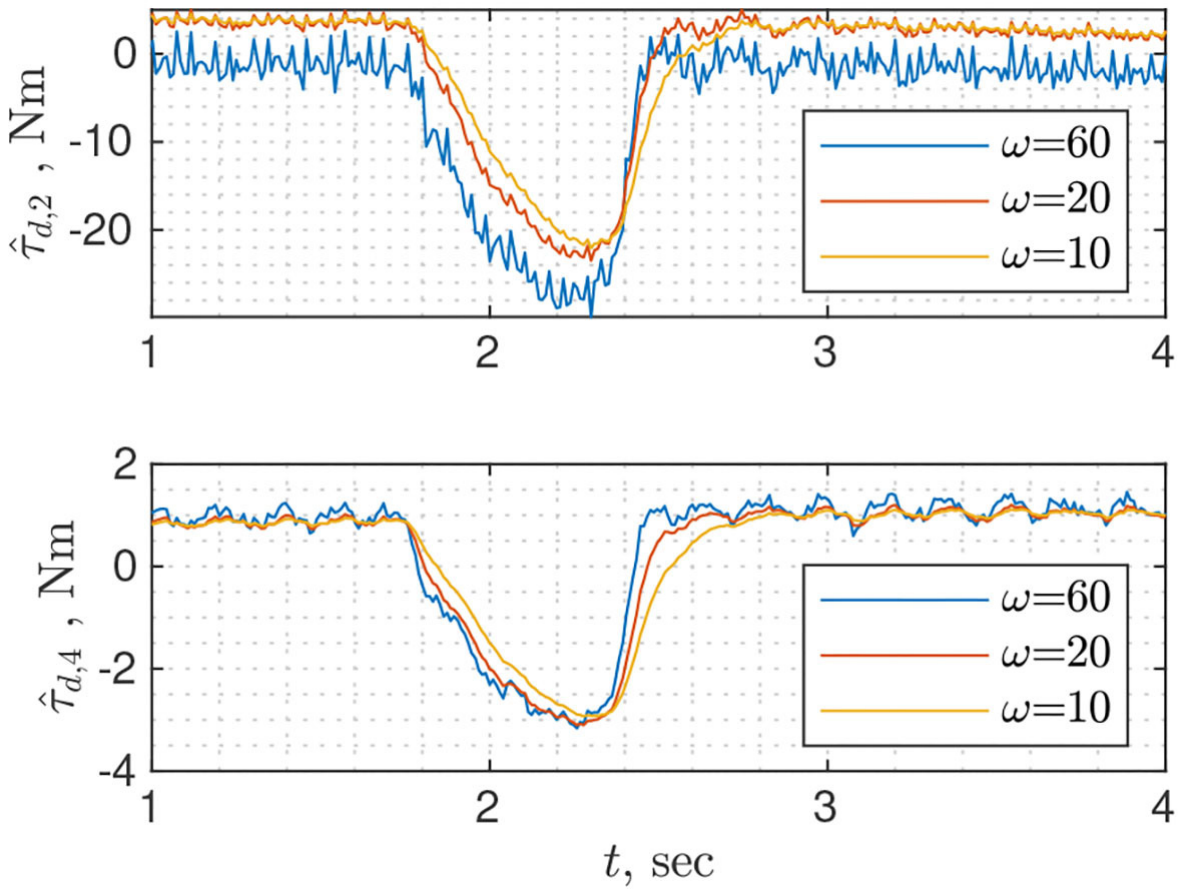
Disturbance torque estimation for joints 2 and 4 using filtered dynamics observer with different cutoff frequencies.

Probably the simplest observer in terms of tuning is the filtered dynamics observer (Equation 35) as it requires tuning the single parameter: cut-off frequency of the low-pass filter. It is identical to classical momentum observer when gain matrix ***L*** is a diagonal with identical entries along the diagonal. The drawback is the lack of flexibility e.g., a cut-off frequency can be well suited for the first joints but can be too high for the rest. Figure 6 shows disturbance torque estimation for three different values of the cut-off frequency. Different behavior of ${\hat{\tau }}_{d,2}$ for higher (ω = 60) and lower frequencies (ω = 10/20) can be explained by high frequency dynamics, which was filtered during the model identification phase.

### 3.4. Detecting Collisions

For collision detection we did two experiments: in one experiment the robot experienced a soft collision with a human, while in another test—a hard collision with a brick. In both cases, manufacturer safety presets were set to the least restricted to achieve fast motions. Model-based estimated disturbance torques were compared with those estimated based on the real and target currents of the UR10e that were computed as

(38) $$\begin{array}{l} {\hat{\tau }}_{d,UR}=diag\left(K\right)\left(I_{target}-I_{real}\right), \end{array}$$

where *K* is the identified vector of drive gain coefficients, *I*_*target*_ and *I*_*real*_ are the vectors of measured and target currents, respectively.

Due to the modeling error and torque/current measurements noise, the estimated disturbance torques do not equal zero during nominal operation conditions (without collision). To detect a collision it is necessary to set upper and lower thresholds on estimated disturbance torque in the absence of collisions ${\hat{\tau }}_{d,ub}$ and ${\hat{\tau }}_{d,lb}$, respectively. When a collision occurs, some of ${\hat{\tau }}_{d,i}$ significantly increases depending on the location of a collision crossing corresponding ${\hat{\tau }}_{d,ub,i}$ or ${\hat{\tau }}_{d,lb,i}$. The static threshold is the easiest approach to detecting collisions; it can be chosen for a specific task or the whole workspace (any task). On the one side, task-specific bounds for a single or several tasks are tighter and result in faster collision detection time. On the other side, if a robot is reprogrammed to execute a new task, then the threshold can be violated in the absence of collision. Thus, for robots that are constantly reprogrammed to execute different tasks, global bounds are more advantageous even though they can be looser and can result in higher detection time.

**Figure 7:**
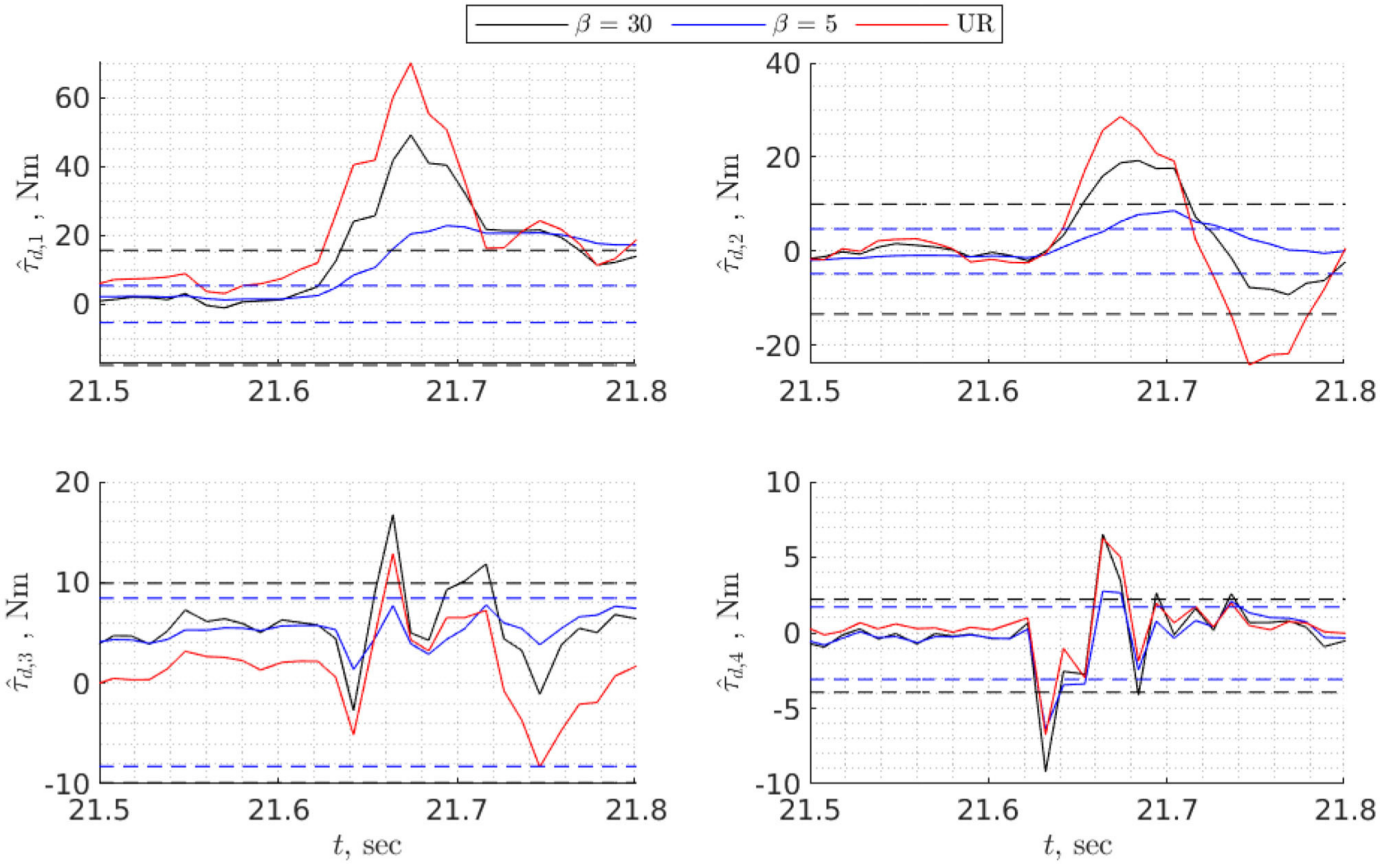
Detecting a hard collision of the UR10e with a brick using static threshold. Thresholds and estimated disturbance torques are shown for two nonlinear disturbance observers with different rate of convergence. The third disturbance torque (in red) is estimated from current measurements of the UR10e. Dashed lines are thresholds associated with specific value of β.

**Figure 8:**
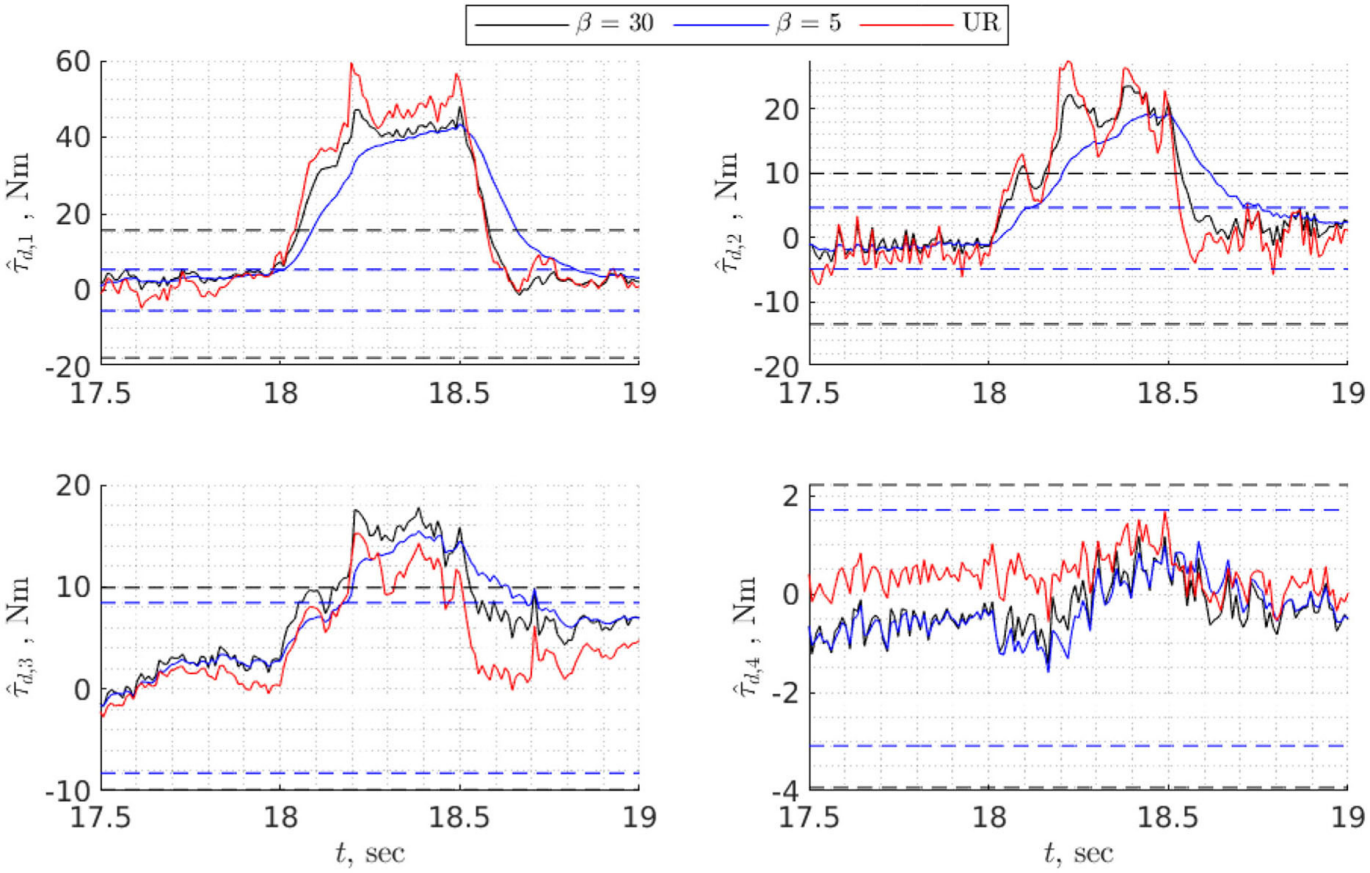
Detecting a soft collision of the UR10e with a human using static threshold. Thresholds and estimated disturbance torques are shown for two nonlinear disturbance observers with different rates of convergence. The third disturbance torque (in red) is estimated from current measurements of the UR10e. Dashed lines are thresholds associated with specific value of β.

To find task-specific thresholds it is necessary to execute a task making sure there is no collision, then estimate disturbance torques and choose bounds that exceed maximum and minimum values of ${\hat{\tau }}_{d,i}$ by 5–10% (**Figure 8**). For global bounds, it is possible to use data from validation or execute several trajectories that cover the whole workspace, then perform the procedure similar to the one for task-specific thresholds. In any case, the bounds will depend on the choice of disturbance observer, particularly on the convergence rate: the higher the convergence rate, the higher the bounds and vice versa (**Figure 8**). However, although for faster convergence rates the bounds are higher, the detection time is shorter, especially for fast collisions (Figure 7).

Both Figures 7, 8 show that collision detection capabilities of the UR10e and model-based collision detection algorithms with β = 30 were identical for hard collisions (Table 4), while for soft collisions UR10e was slightly faster (Table 5). One possible explanation is that the internal control loop of the UR10e is much faster than the rate at which we read generalized coordinates and velocities, and currents which are around 100 Hz. Another explanation is that the convergence rate of the disturbance observer inside the UR10e control system is higher than the convergence rate of the non-linear disturbance observer we use. The behavior of slower disturbance observer with β = 5 is different from the others; for example, in the case of hard collision of the third joint, estimated disturbance torque did not cross thresholds (Figure 7), but overall collision detection time was the same as for β = 30. In the case of soft collision, the observer with β = 5 was the fastest to detect collision (Table 5). In view of this result, the best strategy could be to use several disturbance observers with different convergence rates and static thresholds.

**Table 4:** Identified link parameters.

**Joint no**	**Collision detection time**, *****s*****		
	****β = 30****	****β = 5****	***UR***
1	21.642	21.642	21.642
2	21.654	21.674	21.654
3	21.664	–	21.664
4	21.632	21.632	21.632
Overall	21.632	21.632	21.632

**Table 5:** Identified motor and friction parameters.

**Joint no**	**Collision detection time**, *****s*****		
	****β = 30****	****β = 5****	***UR***
1	18.052	18.02	18.042
2	18.082	18.136	18.072
3	18.178	18.198	18.208
4	–	–	–
Overall	18.052	18.02	18.042

Theoretically, thresholds can be significantly reduced for fast collisions—collisions that have a distinguishingly higher frequency than the motion of robot—by a band-pass filtering, the dynamics of the robot (Ho and Song, 2013; Li et al., 2019). However, in our experiments by applying band-pass filter we were not able to reduce thresholds and to obtain faster collision detection time without getting false positives. A possible explanation is that the trajectory had abrupt changes in the sign of generalized velocities that caused fast changes in the friction torques, therefore the dynamics of the robot had high frequencies.

Another way to reduce the threshold is to use machine learning to predict unmodeled dynamics (Hu and Xiong, 2017). If enough data is provided for training, then neural networks can approximate unmodeled dynamics with a high degree of accuracy. The limitation of this method is that it requires knowledge of the field that engineers may not have, making it suitable for few who are familiar with neural networks.

## 4. Conclusion

This paper has outlined a roadmap for engineers and specialists new to the field, to equip industrial manipulators with collision detection capabilities needed to guarantee workplace safety. The roadmap consists of three main steps: identifying the dynamic model, choosing and tuning the disturbance observer, and detecting collisions based on the output of the disturbance observer. The most important step is model identification because the accurate model allows choosing tighter thresholds, and consequently reducing collision detection time. Accurate dynamic parameter estimation might be time-consuming, especially its trajectory planning phase (depending on the trajectory and the number of parameters, trajectory optimization can take up to 10 h on a usual desktop computer), which is necessary to guarantee persistent excitation. At the cost of model accuracy, the trajectory planning phase can be notably simplified by omitting optimization, and instead use several points connected with fifth-order polynomials. The rest of the steps, unfortunately, cannot be further simplified.

For disturbance estimation, there is a great variety of disturbance observers. If simplicity is the priority, then non-linear disturbance observer or filtered dynamics observer can be chosen as they are tuned with single parameter β and ω, respectively. If more flexibility is required, then a classical momentum observer can be used because it allows prescribing the convergence rate of each joint separately. If better measurement and model noise filtering are required, then the Kalman momentum observer should be used. If finite time convergence is required, then second-order sliding mode observers should be chosen.

As the final step, it is necessary to choose thresholds for estimated disturbance torques in the absence of collisions, those can be chosen regardless of task type or on task-specific basis. Task-specific thresholds are tighter and result in faster detection time, while global bounds are not limited to any task but may result in slower detection time. In both cases, thresholds depend on the convergence rate of the disturbance observer, the faster the observer, the higher the threshold. In our experiments, faster disturbance observers were better at detecting hard collisions, while slower observers—at detecting soft collisions. It can suggest that the use of both slow and fast observer can lead to faster collision detection regardless of the type of collision. To further decrease collision detection time, several authors proposed ways to reduce threshold by using band-pass filtering, neural networks, while others proposed to use time-varying thresholds. Band-pass filtering works well with very fast collisions, depends on the dynamic model (friction model) and the trajectory of robot—in our experiment with a brick band-pass filtering did not decrease collision detection time—the use of neural networks requires a certain background that prevents engineers from using it. Finally, identifying coefficients of the time-varying thresholds involves time consuming joint-wise experiments and do not result in a significant reduction in collision detection time (Sotoudehnejad et al., 2012).

## Data Availability Statement

All datasets presented in this study are included in the article/Supplementary Material.

## Author Contributions

SMa did the literature review, dynamic identification and wrote the paper. SMi implemented disturbance observers library, did experiments with the robot and took part in writing the paper.

## Conflict of Interest

The authors declare that the research was conducted in the absence of any commercial or financial relationships that could be construed as a potential conflict of interest.
